# Supplementary material for: Are carbon emissions trading and green financial instruments synergistic? -Comprehensive quantitative research based on content analysis
Source: PLoS One. 2024 Mar 7;19(3):e0298601. doi: 10.1371/journal.pone.0298601 (PMC10919601; doi:10.1371/journal.pone.0298601)
Supplement: S1 Data — (ZIP) [file pone.0298601.s001.zip › Data/地级市Z_F工作报告.zip!/╔╜╢1⁄2/╝├─╧╩╨/└┤╫╘╣1⁄2╓┌║┼í╛┬φ┐╦╩2╛▌═°í┐_2006.docx]

各位代表：

　　现在，我代表市人民政府向大会作工作报告，请连同《济南市国民经济和社会发展第十一个五年规划纲要(草案)》一并审议，并请各位政协委员和其他列席人员提出意见。

　　一、“十五”时期经济社会发展取得重大成果

　　新世纪的第一个五年，是我市全面建设小康社会取得阶段性重大成果的五年。全市人民以邓小平理论和“三个代表”重要思想为指导，认真落实科学发展观，紧紧围绕“实现新跨越，建设新泉城”奋斗目标，按照“发挥省城优势，发展省会经济”总体思路，解放思想，干事创业，攻坚破难，开拓进取，经济、社会、生态和城乡建设朝着更加协调的方向迈进，省会现代化建设步入新的阶段。

　　(一)结构调整成效明显，综合经济实力显著增强。大力推进经济结构战略性调整，努力转变经济增长方式，国民经济保持快速协调健康发展。初步核算，2005年，全市实现生产总值1876.5亿元，年均增长14.2%，比2000年接近翻一番，人均3916美元。地方财政一般预算收入106亿元，年均增长16.7%，是2000年的2.2倍。固定资产投资累计完成2762亿元，年均增长20.4%，是“九五”时期的2.4倍。社会消费品零售总额807.9亿元，年均增长17.9%，是2000年的2.3倍。金融机构各项存款余额3483.3亿元，贷款余额3259.9亿元，分别是2000年的2.7倍和3倍。三次产业比例由2000年的10：43.9：46.1调整为7.1：46：46.9。工业比重39.1%，比2000年提高3.8个百分点，对经济发展的支撑力逐步增强。固定资产投资率45.7%，比2000年提高13.6个百分点。非公有制经济比重41%，高新技术产值比重30.1%，经济外向度16.2%，比2000年分别提高16个、9.9个和3.7个百分点。收入过亿元企业608家。经济整体素质和竞争力显着提高。

　　(二)区域统筹力度加大，“三农”形势发生积极变化。把破解“三农”问题摆在重中之重位置，初步构筑起以工促农、以城带乡反哺农业新机制。农业综合生产能力明显提高。实施“6521”和“1020”工程，五年各级财政支农投入24.6亿元，是“九五”时期的2倍，2005年粮食总产260.1万吨，依靠科技进步和结构调整推进农业提质增效步伐加快。农村基础设施条件明显改善。村村通油路(水泥路)、通自来水、通客车、通广播电视率分别达到95.8%、62.6%、99.3%和100%。农村生态环境明显改善。荒山造林、退耕还林、平原绿化、绿色通道建设、小流域治理、秸秆利用力度加大，生态农业取得积极进展。不断加强农村公共服务能力。教育、卫生、文化条件得到改善，农村社会保障范围不断扩大，174.4万农民参加新型合作医疗。大力推进农村税费改革，全部免征农业税，农民负担明显减轻，累计16.8万人实现脱贫。统筹规划，分类指导，完善政策，推进基础设施向农村延伸，县域经济发展活力显着增强。

　　(三)城市功能更加完善，省城发展框架全面展开。按照“东拓、西进、南控、北跨、中疏”空间战略和“新区开发、老城提升、两翼展开、整体推进”发展思路，加快推进城市建设。高起点实施城市总体规划修编和控制性详规编制，规划的科学性、前瞻性、指导性全面增强。高标准实施“十大工程”建设，完成城建投资850亿元，是“九五”时期的2.2倍。东区主干路网基本形成，西区大学科技园初具规模，“三网一枢纽”交通构架形成，航空运力不断增强，电力供应保持稳定，通讯网络广泛覆盖，老城面貌部分发生质的变化。高效能推进城市管理，城乡环境综合整治成效显着，居民生活环境和城市生态得到较大改善。加大保泉力度，实现泉水两年多来持续喷涌。2005年，全市城镇化率达到56%。城市人均道路15.8平方米、人均公共绿地9平方米，比2000年分别提高25.4%和26.8%。城市建成区绿化覆盖率、污水处理率、燃气普及率、集中供热普及率、垃圾无害化处理率、空气质量良好以上天数比率分别达到39.6%、54.5%、97.2%、46%、99.6%和72%，比2000年分别提高3.5、9.6、6.5、10、13. 5和4.8个百分点。城建运行体制不断完善，初步建立起科学民主决策的规划管理体制，高效廉洁运作的重点工程管理体制，自求平衡、滚动发展、良性循环的城市运营体制，城市综合执法和管理重心下移的城区管理体制。土地市场管理步入法制化、市场化轨道。

　　(四)各项改革综合推进，体制环境更具发展活力。围绕推进市场化进程，优化发展环境，增强发展活力，大力推进各项改革。着力完善国有资产监管和授权经营制度，上市公司积淀的矛盾和问题得到化解，纳入国资委监管的255户企业完成改制126户。立足发挥各方潜能，着力推进财税体制、在地统计制度和土地收益制度改革，理顺市与县(市)区及高新区事权财权关系。以提高行政效能为目标，推进政府机构和行政审批制度改革，精简职能错位交叉机构29个，依法取消行政许可主体27个、行政许可事项695项、收费项目252项。重大决策事项征询听证和民主评议政府制度全面推进，行政决策执行监督制度不断完善。实施政府资金管理制度、资产管理制度、政府采购制度和国库集中支付制度改革，支出行为更加规范，行政成本逐步降低。实施政府投融资体制改革，初步构筑起项目法人责权明确、融资投资行为规范、投入产出自求平衡、政府债务风险化解的重点工程建设保障机制。

　　(五)对外开放步伐加快，城市开放程度不断提高。把握入世机遇，全方位推进对外开放。招商引资成效显着。坚持“市外即是外”，大力度吸引域外资金参与园区建设、产业发展、国企改革、结构调整，五年引进资金1020亿元，在济世界500强企业已达22家。对外贸易长足发展。国家级出口加工区封关运行，国际航线不断增加，出口门类不断增多，高附加值的机电、高新技术产品出口比重加大。五年进出口总额118.2亿美元，是“九五”时期的2.3倍。开放领域不断拓展。国有企业、现代服务业、房地产业利用外资较快增长，公用事业及金融业利用外资取得突破。对外合作逐步增强。境外投资和项目规模不断扩大，对外承包和劳务合作营业额累计8.1亿美元，年均增长69.9%，是“九五”时期的11.5倍。经济、技术、教育、文化领域对外交流合作愈加紧密，总体对外开放水平明显提高。

　　(六)社会事业加快发展，公共服务能力不断加强。注重社会与经济协调发展，保持社会事业投入较快增长。全社会研发投入占生产总值比重比2000年翻一番，五年取得重要科技成果1000余项，同比增长25%，科技成果转化率达到30%。高中和高等教育毛入学率分别为77%和27.5%，比2000年分别提高19个和10.5个百分点。普通高校在校生总数、全市人才总量分别达到38万人、61万人，分别是2000年的3.4倍和1.4倍。文艺创作和文物保护取得新成绩，群众文化活动更加丰富。成功战胜非典疫情，疾病防控救治能力得到增强。群众体育跨入全国先进行列，竞技体育有了新的突破。五年安置城镇就业再就业40.7万人，城镇登记失业率控制在4%以内。社会保障制度逐步健全。市财政五年累计投入社会保障资金39.6亿元，全市各项社会保险征缴收入149亿元，分别是“九五”时期的5倍和2倍，基本实现应保尽保。推行住房公积金和廉租房制度，帮助2万多名城镇职工和部分困难家庭解决住房问题。人口和计划生育工作持续加强，低生育水平保持稳定。新闻出版、广播电视、社会科学、档案史志、外事侨务、民族宗教、妇女儿童、老龄、残疾人等事业都有新发展。

　　(七)消费结构逐步改善，人民生活水平明显提高。坚持以人为本执政理念，高度关注人民生活，努力使人民群众从经济增长中得到更多实惠。居民收入持续增加。2005年城市居民人均可支配收入12310元，年均增长10.3%。农村居民人均纯收入4812元，年均增长9.6%。居民消费价格指数101.1%，物价保持基本稳定。城乡居民储蓄存款1024.4亿元，年均增长17.2%。消费结构不断升级。

　　城市和农村居民恩格尔系数分别为33%和39.1%，比2000年分别下降1.6和4.4个百分点。百户城市居民拥有汽车5.4辆、电脑54.2台、移动电话145.2部，比2000年分别增长17倍、1.7倍和4倍。居住环境明显改善。城市居民人均住房使用面积19.8平方米，农村居民人均生活用房面积33.8平方米，比2000年分别增加3.3和5.2平方米。生活方式转变加快。伴随收入水平提高，城市居民由追求基本物质需求向精神、文化需求转变，旅游、教育、健身、艺术等成为新的消费需求，人口平均预期寿命由72岁提高到74.4岁。

　　(八)社会文明不断提升，和谐社会建设稳步推进。从解决事关群众切身利益的突出问题入手，推进“和谐济南”建设。民主法制建设全面加强。坚持依法治市，五年提请市人大常委会审议通过地方性法规(草案)26件，制定政府规章49件，办理人大代表建议1820件、政协提案2502件，民主科学决策和依法行政水平进一步提高；法律服务体系日益健全，社区建设稳步推进，全民法律意识和法制观念不断增强。文明城市创建扎实有效。深入开展文明创建和为群众办实事活动，大力弘扬“诚信、创新、和谐”城市精神，逐步形成健康稳定、奋发向上的社会氛围；国防教育、国防动员、人民防空得到加强，荣获全国双拥模范城“五连冠”。“平安济南”建设成效明显。强化社会治安综合治理，群众安全感达到92.5%；加强安全生产专项整治，安全事故发生起数和伤亡人数大幅下降。加强食品药品市场监管，“放心工程”取得明显成效。加大腐败防治力度，加强监察审计，强化廉政建设和社会监督，廉政建设和反腐败斗争工作呈现良好发展态势，人民群众满意度有了新的提高。

　　各位代表！五年的巨大变化，凝聚着全市人民的智慧和汗水。五年中，我们遇到的困难比预料的大，取得的成绩比预想的好。面对“入世”带来的冲击，经济体制转轨和社会转型加快、矛盾增多的复杂局面，非典、禽流感等重大疫情及自然灾害的威胁，全市上下同心同德，奋力拼搏，突破一个又一个难点，圆满完成“十五”计划。五年来的发展和进步，是省委、省政府和市委正确领导的结果，是全市人民艰苦奋斗、开拓进取的结果，是全市上上下下、方方面面团结一致、共同努力的结果。在此，我代表市政府向辛勤工作在各行各业的全体市民，向给予政府工作大力支持的各级人大代表、政协委员、各民主党派、工商联、各界人士，向关心支持地方工作的人民解放军驻济部队、武警部队官兵，中央、省驻济单位，向为济南发展做出贡献的港澳台同胞、海外侨胞、国内外朋友，表示崇高的敬意和诚挚的感谢！

　　回顾“十五”历程，我们在创造巨大物质财富的同时，也积累了宝贵精神财富，这就是：坚持以科学发展观统领经济社会发展全局。突出发展第一要务，抢抓发展机遇，加快发展步伐，转变发展模式，提高发展质量，努力在发展中解决各类矛盾，不断推进经济社会发展迈上新台阶。坚持以人为本执政理念。着力解决好事关人民群众切身利益的突出问题，正确处理改革发展稳定的关系，更加注重社会公平，努力促进社会和谐。坚持“发挥省城优势，发展省会经济”总体思路。实施“六个突破、六个互动”推进方式，不断创新探索，努力走出一条符合科学发展规律、符合济南实际的发展道路。坚持用改革、市场和创新的办法解决问题。把握经济和社会发展内在规律，加快政府职能转变和市场化进程，构建有利于科学发展的经济、行政、市场环境和城市规划、建设、管理秩序。坚持把破解深层次矛盾作为实现长远发展的着力点。把握发展的阶段特征，抓住制约发展的突出矛盾和关键问题实施突破，不断提高发展的稳定性和协调性。坚持求真务实、真抓实干。一切从实际出发，体现科学、理性、务实精神，出实招、办实事、求实效，埋头苦干，攻坚破难，不断推进经济社会又快又好发展。这些认识和体会，是我们仍将继续坚持和完善的重要无形资产。

　　各位代表，“十五”经济社会发展成果令人鼓舞，前进中也面临不少矛盾和问题。一是深层次结构性矛盾仍较突出。工业经济、非公有制经济、县域经济、固定资产投资比重和经济外向度仍然偏低，城市综合竞争力有待增强。二是经济增长方式仍较粗放。生产集约化程度不高，自主创新能力不强，生态环境比较脆弱，经济发展与资源环境的矛盾比较突出。三是促进社会和谐发展的保障能力较弱。社会事业投入不足，就业和社会保障压力较大，不稳定不安全因素仍然存在。四是城乡区域发展不协调。区域经济发展不平衡，城乡二元结构矛盾仍较突出，公共产品城乡覆盖程度差别较大，缩小区域差距的任务艰巨。五是体制机制问题亟待解决。经济体制总体不活，市场配置资源的基础性作用远未充分发挥，生产力发展仍面临诸多体制机制障碍。六是政府公共服务能力有待提高。政府职能转变还不适应市场经济发展的要求，促进科学和谐发展的能力、应对突发事件的能力、社会管理和公共服务的能力有待提高。这些事关全局的大问题，我们一定高度重视，认真对待，努力解决。

　　二、努力实现“十一五”时期经济社会又快又好发展

　　“十一五”是我市加快现代化进程的重要时期。经过改革开放以来的积蓄发展，全市综合经济实力迈上新的台阶，经济社会发展站在新的起点，工业化、城镇化、现代化进入新的阶段。我们既面临产业结构加快调整、消费结构加快升级、城镇化步伐加快推进、社会结构加快转型、体制机制加快完善以及承办十一届全运会等六大发展机遇，也面对区域竞争日趋激烈、发展与资源生态矛盾尖锐、城乡协调发展任务艰巨、就业与保障压力仍较突出、各类社会矛盾凸显以及老城提升更为艰难等六个方面的挑战。纵观国内外宏观环境变化趋向，总体上有利于实现又快又好发展。我们有信心、有责任，站在新的历史起点，瞄准新的发展目标，努力开创省会现代化建设新局面。

　　“十一五”时期全市经济社会发展的指导思想是：以邓小平理论和“三个代表”重要思想为指导，深入贯彻党的十六大和十六届五中全会精神，以科学发展观为统领，以构建和谐社会为目标，深入实践“发挥省城优势，发展省会经济”总体思路，突出发展第一要务，加快社会主义新农村建设，推进经济结构战略调整，大力实施新型工业强市、科教兴市和可持续发展三大战略，切实抓好自主创新、完善体制机制和增强发展后劲三大关键，着力实施转变增长方式、提升城市功能、扩大对外开放和促进协调发展四大突破，努力推动产业实力更加雄厚、科技教育更加发达、服务功能更加完善、人民生活更加殷实，加快建设具有深厚历史文化底蕴、独特自然风貌、浓郁现代气息的省会城市和区域中心城市。

　　“十一五”时期全市发展的总体目标是：

　　——经济实力实现新的跨越。国民经济保持快速协调健康发展，到2010年，全市生产总值达到3500亿元，年均增长13%，人均6800美元。地方财政一般预算收入达到210亿元，年均增长15%。社会消费品零售总额达到1550亿元，年均增长14%。固定资产投资累计完成7200亿元，年均增长18%。产业结构进一步优化，三次产业比例调整为4.5∶50∶45.5。

　　——经济增长方式明显转变。自主创新能力显着增强，全社会研发投入占生产总值比重提高到2.5%左右，高新技术产值比重提高到40%以上。单位生产总值能源消耗比“十五”期末降低22%左右、耗水量降低14.5%，全市工业用水重复利用率达到74%。

　　——城市服务功能全面提升。城市基础设施更加完善，综合服务功能不断增强，城市面貌和生态环境有较大改善。中心城区建成区面积达到330平方公里，人口达到340万人。城市人均道路面积达到17平方米，城市建成区绿化覆盖率达到43%，全市森林覆盖率达到28%。正常降水条件下，泉群保持常年喷涌。城镇体系更加合理完善，城镇化率提高到65%。

　　——改革开放取得新的突破。基本完成国有经济战略性调整，非公有制经济比重提高到60%。重点领域改革取得突破性进展，建立起比较完善的社会主义市场经济体制。经济国际化程度显着提高，实际利用外资和外贸出口年均增长分别为20%和15%，五年累计实际利用外资45亿美元，2010年外贸出口达到36亿美元。

　　——协调发展能力显着增强。全市基本普及高中阶段教育，高等教育毛入学率提高到50%。公共卫生、文化、体育等服务体系更加完善。全市户籍总人口达到640万，人口自然增长率控制在5‰左右。经济发展与人口、资源、环境相协调，城市中心区、近郊区、远郊区实现互动发展，形成城乡统筹协调发展新格局。

　　——社会和谐水平明显提高。政治昌明廉洁，居民生活富裕，社会治安良好，安全生产平稳，依法行政能力进一步增强，市民综合素质和社会文明程度大幅提升。城乡就业和社会保障水平进一步提高，城镇登记失业率控制在4.2%以内。城市居民人均可支配收入和农村居民人均纯收入年均增长8%，价格总水平基本稳定，城乡恩格尔系数分别降到30%和37%。

　　“十一五”时期全市经济社会发展的主要任务是：

　　(一)大力推进社会主义新农村建设。按照生产发展、生活宽裕、乡风文明、村容整洁、管理民主的要求，坚持工业反哺农业、城市支持农村和多予少取放活的方针，加快农业产业化、农村工业化和城镇化进程，完善促进农民增收长效机制，扎实推进社会主义新农村建设。一是大力发展现代农业。合理划分区域功能，建设环城都市农业、南部生态农业、沿黄高效农业等特色农业区。大力推进农业结构调整，加快推进农业科技进步，提高农业产业化、标准化、国际化水平，着力构筑区域化布局、专业化生产、规模化经营、社会化服务、企业化管理的现代农业格局。二是着力培育新型农民。把培养有文化、懂技术、会经营的新型农民，作为新农村建设最本质、最核心的任务。以加强精神文明教育、农村政策教育、职业教育和技能培训为重点，增强农民就业创业能力，加快农村劳动力转移步伐，推进农民生产生活方式转变。三是提高农村公共服务水平。扩大公共财政对农村覆盖范围，落实各项扶持和补贴政策，加大财政转移支付力度，增加对农村公共设施和农业基础设施投入，继续推进产业扶贫。搞好乡村建设规划，保护生态环境，加快改善农村面貌。四是深化农村综合改革。按照依法、自愿、有偿原则，促进土地合理流转，大力推进乡镇机构、农村义务教育、县乡财政体制改革，逐步消除阻碍城乡协调发展的体制机制障碍。

　　(二)持续推进产业结构优化升级。以提高自主创新能力为核心，以三次产业协调发展为目标，形成以高新技术产业为先导、先进制造业为支撑、服务业全面提升的现代产业格局。一是坚持新型工业强市战略。坚定不移地把工业放在优先发展地位，以信息化带动工业化，以工业化促进信息化，大力实施“6511”工程，突出发展交通设备、电子信息、冶金钢铁、石化化纤、机械装备、食品药品六大产业集群，积极培育壮大新兴产业，强化工业园区带动功能，构筑富有竞争力和可持续发展能力的工业体系。二是加快发展高新技术产业。坚持自主创新与技术引进相结合，大力发展信息、生物、新材料、新能源等新兴产业，广泛应用高新技术和先进适用技术改造提升传统产业，努力培养形成一批具有较强国际竞争力、自主知识产权和知名品牌的优势企业。三是发展壮大建筑业。加快建立统一开放、竞争有序、适应市场经济发展要求的建筑产业体系、市场体系和企业运行机制。实施大建筑业、大企业和大市场战略，显着增强建筑业的综合竞争力。四是创造服务业发展新优势。强化区域服务功能，全面推进生产性、生活性和公共性服务业发展提升，积极推进福利型、公益型、事业型三产机构向经营型转变，突出发展金融、物流、旅游、会展等现代服务业，积极发展信息、中介、房地产、社区服务等新兴服务业，运用先进经营方式和信息技术，着力提升商贸、餐饮、交通运输等传统服务业，构筑产业层次较高、布局合理、特色鲜明、竞争力强的区域性服务业中心。

　　(三)加快省会城市现代化建设。突出城市特色，加强规划建设管理，大力提升省会城市现代化水平。一是增强城市承载能力。按照系统化、网络化、现代化标准，适度超前建设综合交通、能源、信息通信、供排水、城市生态及城市安全六大系统。全面推进现代立体交通体系建设，发展快速公交系统，提高区域交通通达性；着力构筑稳定、经济、清洁的能源供应体系，保障经济社会发展需求；加快整合提升信息资源，形成覆盖全市的高速信息网络；大力提高供排水能力和质量，构筑安全可靠的供排水体系；加快构筑生态保护与恢复体系，形成城市可持续发展的生态支撑系统；建立完善防灾减灾综合体系，确保城市安全运行。二是加快新区开发建设。坚持以新区开发反哺老城提升，有序加快新区建设步伐。以承办第十一届全运会为契机，加快建设奥体中心和政务中心，打造以政务、文化、体育为主的燕山新区。实施张庄机场搬迁，打造以物流、商务为主的腊山新区。加快孙村片区、出口加工区、力诺工业园建设，打造以高新技术产业、高附加值制造业为主的东部新区。加快大学科技园区建设，打造以高等教育、科研、旅游休闲为主的西部新区。三是加快老城改造提升。突出历史文化名城和独特自然景观的保护，完善旧改推进机制，加快旧城改造步伐，加强城乡综合整治，引导规范旧村居改造，突出“山、泉、湖、河、城”浑然一体、特点鲜明的泉城风貌。四是完善城市管理体系。加快完善规划体系，实现中心城区控制性详细规划全覆盖。完善“两级政府、三级管理、四级网络”城市管理体制，加强城市管理综合执法，着力解决集贸市场退路难和中心区停车难等问题。完善城建投融资体系，保障建设资金良性循环。完善城建项目运行体系，确保城市建设工程顺利实施。

　　(四)努力促进城乡区域协调发展。以城乡一体化为目标，明确不同区域功能定位，促进城镇化健康发展，形成城乡协调发展新格局。一是以“四沿”为支撑加快中心城区发展。实施沿经十路、沿铁路线、沿小清河、沿黄河“四沿”带动，推动重点片区建设，形成连接东西、辐射南北的经济、社会、生态、城市发展带。二是以“三区”互动为重点推进城乡协调发展。着力提升中心区、做强近郊区、突破远郊区。推动中心区功能外延，努力形成相互促进、互利共赢、优势互补、共同发展新格局。加快以路桥建设、生态保护和新区开发为重点的北跨步伐。三是以突出生态功能为基础实施南部山区保护开发。以生态改善和农民增收为目标，强化南部山区生态环境保护，发挥自然景观、人文资源和特色农副产品优势，实现生态建设产业化、产业发展生态化。四是以“四级”城镇体系为依托推进城镇化进程。以拓展中心城市发展空间和培育中等城市为重点，逐步形成以中心城市为核心、大中小城镇有机结合、规模序列完善、职能分工互补、空间布局合理的城镇体系。五是以市场配置资源为主导加快构筑济南城市圈。强化区域协作，增强服务区域发展的能力，着力构建优势互补、资源共享、信息互通、产业互动、合作共赢的良性机制，加快区域人才市场、交通网络、信息网络、商贸物流一体化进程。

　　(五)继续深化改革和扩大开放。深化完善社会主义市场经济体制，加快与国际经济全面接轨，不断增强经济社会发展的动力和活力。一是深化行政管理体制改革。继续完善政府资金管理、行政资产管理、行政事务管理、行政审批制度、政务公开制度等项改革，分类推进事业单位改革。强化社会管理和公共服务职能，提高行政效率和服务水平。二是继续推进和完善综合配套改革。大力推进国有企业改革改制，健全现代产权制度和国有资产管理体制，完善国有资本有进有退、合理流动机制。进一步放开市政公用市场，改革价格管理机制，推进投资多元化和运营市场化。深化财税体制、投资体制改革，规范资金管理，提高资金效益。加强现代市场体系建设，重点发展资本、劳动力、土地、产权等要素市场，切实加强市场监管，创造良好市场环境。三是全面提高对外开放水平。突出外向带动，以重点领域和项目为依托，强化载体建设，创新招商方式，优化发展环境，积极扩大利用外资领域和规模。优化对外贸易结构，扩大高附加值产品出口，培育壮大一批具有国际竞争力的出口创汇型企业。四是大力加强经济技术合作。鼓励支持有条件的企业“走出去”，开拓国际市场，用好两种资源。充分发挥各界对外开放积极性，全方位、多层次、宽领域开展对外交流合作。扩大对内开放，搞好区域合作，积极吸引国内资金。五是大力发展非公有制经济。着力营造平等竞争的法制环境、政策环境和市场环境，依法保护非公有制经济合法权益和私有财产。进一步放宽市场准入，鼓励非公经济进入公用事业、社会事业、国有经济结构调整等领域，引导支持民营企业做大做强。

　　(六)着力提升城市自主创新能力。坚持科教兴市和人才强市战略，建设创新型城市，提高城市核心竞争力。一是加快推进科技创新。完善以企业为主体、市场为导向、品牌为目标、产学研相结合的技术创新体系，加强原始创新、集成创新和引进消化吸收再创新，努力实现关键核心技术的集成突破。积极推进科技创新平台建设，建立完善知识产权保护、风险投资、技术产权化三项制度，加大科技引导投入，构建自主创新制度支撑。二是优先发展教育事业。加快构筑多元化全民教育体系，积极调整教育结构，大力整合教育资源，全面推进素质教育，实现基础教育巩固提高、均衡发展。以成才途径多元化和促进就业为导向，以技能型实用人才培养和农村职业教育为重点，加快发展职业教育。充分发挥省会城市教育资源优势，积极发展高等教育。三是着力提升文化竞争力。推动文化与经济融合发展，继承挖掘历史文化，吸收弘扬先进文化，深化文化体制改革，繁荣文化事业，发展文化产业，提高全民文化素质，推进自然科学、哲学和社会科学繁荣发展、交融互补，增强省会现代化建设的凝聚力。四是努力构筑人才高地。加强人才资源建设，调整优化人才结构，努力抓好人才培养、引进和使用三个环节，大力实施行政人才能力提升、企业家培养、专业人才开发、高技能人才振兴和农村实用人才培训五大工程。加大高层次人才引进力度，深化企事业单位用人制度和分配制度改革，建立充满生机和活力的人才激励机制。五是推进国民经济和社会信息化。围绕建设区域性信息中心，加强信息技术在社会各领域的广泛应用，培育繁荣信息服务市场，大力发展软件业与信息服务业，加快推进产业、商务、政务和社会公共服务信息化建设步伐。

　　(七)全面推进城市可持续发展。全面落实节约资源和环境保护的基本国策，促进人与自然和谐发展，按照“五节一循环”要求，加快构建资源节约型、环境友好型社会。一是保护节约利用资源。加强土地资源优化配置。按照优化结构、盘活存量、节约挖潜和实行最严格耕地保护政策的要求，大力推进土地开发整理复垦，显着提高土地投资强度和集约利用水平。严格保护和合理开发利用水资源。加强水资源管理和供水管理，努力降低水资源消耗，推进城市污水资源化处理，加快建设节水型社会。保护和高效开发利用矿产资源。加强矿产资源治理整顿，规范矿产市场秩序，促进有序开发、综合利用和分类管理。加大能源节约力度。制定实施严格的产品消耗标准，坚持开发与节约并举，积极发展新能源，建立多元能源结构，最大限度降低综合能耗。二是加快发展循环经济。把发展循环经济作为转变经济增长方式的中心环节，鼓励发展资源节约、综合利用、废旧物资回收等生态产业，加大以节能降耗为主要内容的结构调整和技术改造力度，逐步形成低投入、低消耗、低排放、高效率的节约型增长方式。坚持政策导向、价格调控和科技支持，推动产业循环式组合、企业循环式生产、资源循环式利用。三是加大污染防治力度。采取综合措施搞好源头防治和污染治理。全面加强大气、水、固体废弃物和声、光、电磁辐射等污染防治，严格饮用水源地保护，全面提升城乡环境质量。四是加强生态环境建设。完善生态功能区划，突出主导功能，合理调整发展模式和发展重点，建设绿色生态体系，防治土地沙化，加快形成城乡一体的大绿化格局，努力构筑促进可持续发展的生态系统。

　　(八)切实解决涉及群众利益问题。牢固树立以人为本的发展理念，坚持以民为先、以民为重，从解决群众最关心、最直接、最现实问题入手，切实让广大人民群众享受到改革发展的成果。一是加快改善农民生活条件。认真落实“两免一补”政策，加快推进农村免费义务教育。普及新型农村合作医疗制度，逐步提高财政补助标准。加快推进农村饮水安全工程，实现农村自来水村村通。实施农村电网改造工程，积极推广太阳能、风能、沼气生态能源的综合利用。二是千方百计扩大就业。把扩大就业放在与经济发展同等重要的位置，实施积极的就业政策，优化就业环境，完善促进就业的长效机制，鼓励劳动者自谋职业和自主创业，关注在济流动人口生存发展，不断完善再就业援助制度。五年安排城镇就业再就业50万人，转移农村劳动力40万人。三是努力提高社会保障水平。加快完善与经济发展相适应、覆盖城乡的社会保障体系，进一步提高城镇职工保障水平，探索建立农村社会保险制度。重点解决好社会困难群体保障问题，提高城镇居民最低生活保障标准，建立农村居民最低生活保障制度，对农村五保户实行财政集中供养。健全困难群众医疗、教育、住房等专项救助制度，大力发展社会福利和慈善事业，加快老年服务、残疾人服务等各类福利设施建设步伐。四是切实加强人民群众医疗健康保障。深化医疗卫生体制改革，加大公共卫生投入，加强乡镇卫生院和社区卫生服务基础建设，增强处置突发公共卫生事件应急能力，加强食品药品市场监管，切实保障人民群众身体健康和生命安全。五是保持物价基本稳定。切实加强事关城乡人民生活的价格监管，重点规范农资、医疗和教育价格秩序。

　　(九)积极推进“和谐济南”建设。按照民主法制、公平正义、诚信友爱、充满活力、安定有序、人与自然和谐相处的要求，扎实推进“和谐济南”建设。一是加强民主法制建设。坚持依法治市和依法行政，推进政务公开，健全权力运行监督机制，提高民主科学决策水平。坚持标本兼治、综合治理、惩防并举、重在预防的方针，加强惩治和预防腐败体系建设，抓好从源头上预防腐败工作，进一步加强廉政建设。完善民主管理制度，发展基层民主，规范行政行为，加强社区建设，充分发挥社会组织在三大文明建设中的重要作用。二是继续推进平安济南建设。完善防范自然灾害、事故灾难、公共卫生事件、社会安全事件的应急体系，提高政府保障公共安全和处置公共事件的能力，进一步强化社会治安综合治理，完善社会利益和社会纠纷调处机制，切实维护人民生命财产安全。三是合理调节收入分配。注重社会公平，加强政策调节，和谐劳动关系，维护劳动者合法权益，积极增加低收入群体收入，努力扩大中等收入群体，严厉打击非法收入，重视解决部分社会成员收入差距过分扩大问题。四是进一步繁荣发展各项社会事业。构建大文化格局，搞好重点文化设施建设，继续繁荣文艺创作和群众文化，逐步形成覆盖全社会的公共文化服务体系。大力发展体育事业，推进全民健身活动，培植竞技体育新优势。加强人口管理和调控，继续改善人口结构。重视民族事业，发展民族经济。五是加强“文明济南”和“诚信济南”建设。深入开展文明城市创建活动，弘扬“诚信、创新、和谐”城市精神，提高全民思想道德水平。按照政府引导、市场运作、法制约束、配套联动的思路，全面推进社会信用体系建设，强化公民信用意识，加大社会失信成本，努力为经济社会发展提供良好的信用环境。

　　三、全力开创“十一五”开局之年工作新局面

　　2006年是实施“十一五”规划第一年。做好今年各项工作，对“十一五”开好局、起好步具有重要意义。今年经济社会发展主要预期目标是：生产总值增长14%，单位生产总值能耗降低5%；固定资产投资增长30%；地方财政一般预算收入增长15%；社会消费品零售总额增长15%；外贸出口增长18%，实际利用外资增长25%；城市居民人均可支配收入增长9%，农村居民人均纯收入增长9%；城镇登记失业率控制在4.1%以内；人口自然增长率控制在5.9‰以内。重点抓好十项工作：

　　(一)继续完善反哺农业机制，实现新农村建设良好开局。坚持规划引导，改革推动，政策支持，扎实推进社会主义新农村建设。一是加快制订新农村建设规划。坚持以发展农村生产力为中心任务，统筹规划农村经济、政治、文化、社会建设，因地制宜、科学论证，尊重农民意愿，抓好试点示范，让农民真正受益。二是积极推进农业结构调整。在确保粮食安全“三条底线”前提下，放手调整农业结构，做强畜牧业、做优蔬菜业、做大林果(苗木花卉)业，不断提高农业综合生产能力。继续实施“双十工程”，扩大主导产业规模，大力提高农业产业化水平。三是促进农民持续增收。继续实施“6521”工程。着力抓好五大农民培训工程，突出引导农村富余劳动力向非农产业转移，力争转移劳动力突破16万人。多渠道开拓农产品市场，加强粮食、农资流通及价格监管。四是做好村镇规划建设。按照科学合理布局、集约利用土地、提升群众生活质量的思路，调整完善小城镇建设和村庄改造规划，深入实施“1020”工程，加快小城镇道路、供水、供电、通讯等基础设施建设步伐，提高小城镇综合承载能力。加强农村公共卫生建设，完善动物疫病三级防治体系，推进文明生态村创建活动深入开展。五是深化农村综合改革。继续落实“两免三补”等农村各项惠农政策，防止农民负担反弹，搞好农村综合改革试点，基本完成乡镇机构、农村义务教育、县乡财政体制改革。

　　(二)坚持增量和集群带动，促进二产优化结构壮大实力。以提高自主创新能力，推动增长方式转变为重点，增强工业经济综合实力。一是持续加大工业投入。以产业政策为导向，按照“三个一批”要求，推动骨干企业扩能改造、产业链条延伸提升、重点园区壮大规模。切实加强项目调度和服务，推动重点项目早日开工建设、竣工达产。力争工业投资占固定资产投资的比重突破50%。二是加快产业结构调整。按照龙头企业、产业链、产业群、产业基地的发展方向，加快推进六大产业群发展，壮大规模实力，增强带动功能。加快推进高技术产业和节能、环保、新能源等新兴产业发展。三是壮大园区产业规模。继续加强园区建设，显着提高园区承载力。把握产业定位，突出园区特色，加大招商引资力度，努力提高产业集聚度和集约用地水平，推动各类园区尽快形成规模效益。四是高度重视品牌打造。以骨干企业为重点，努力提高自主创新能力，在关键领域形成一批具有自主知识产权的核心专利和技术标准，打造有比较优势的产品品牌。五是加快增长方式转变。坚持节约发展、清洁发展、安全发展，大力推进以节能降耗为目标的技术改造，开发和推广资源综合利用、资源节约、资源替代等先进技术，强制淘汰落后技术工艺，积极探索循环经济有效形式。六是推动建筑业加快发展。适应大建设大发展需求，推进建筑业拓展产业领域，提高施工能力，确保工程质量，规范市场秩序，促进优势建筑企业做大做强，加快培育新的经济增长点。

　　(三)繁荣发展现代服务业，提升第三产业层次水平。发挥省城资源优势，优化服务业内部结构，推进服务业加快发展。一是加快发展现代物流业。完善物流产业发展规划，畅通综合交通运输网络，加快担山物流园区建设步伐。对现有物流园区、企业进行挖潜改造，最大限度提高服务能力。二是积极发展金融业。大力推进区域金融中心建设，健全完善组织、市场、监管、诚信体系，优化金融生态环境，引进中外银行、保险、证券等金融机构法人总部、地区总部，大力推动金融资源优化整合，增加金融服务品种。三是做大做强文化产业。加快推进文化体制改革和文化资源整合，完善准入机制，鼓励支持非公有资本以多种方式进入政策许可的文化产业领域，重点发展新闻出版、广播影视、旅游会展和体育产业，培育一批特色鲜明、影响力较强的文化企业，精心承办山东文化产业博览会、第三届信博会等一批重大展会。四是大力发展新兴服务业。着力推进信息服务产业化，加强信息技术在全社会的广泛应用。加快培育引进发展各类中介组织，规范中介组织市场行为。丰富繁荣社区服务业，着力提升社区服务社会化、网络化水平。五是加快房地产业发展。引导住宅类房地产优化供给结构，搞好中低档商品房和经济适用房建设，鼓励商务、金融、科技以及工业类房地产开发，为发展总部经济、楼宇经济提供载体，促进房地产业快速发展。六是提升传统服务业。依托服务业发展规划引导合理布局，推广现代经营方式和信息技术，大力引进国内外知名超市、购物中心、配送中心、连锁店，显着提高传统服务业档次水平。

　　(四)加快新区开发与老城提升，不断提高城市承载力。将科学发展理念贯穿到城市规划建设管理运营的各个方面，努力构建和谐优美的人居环境。一是完善控制性规划体系。先期完成建设意向集中、基础条件成熟的重点片区控制性详规的编制。二是加快新区开发步伐。坚持大规划、大整合、大建设、大发展的运作方式，沿东、西、北三个方向全面展开，整体推进东、西区开发和北跨发展，加快实施奥体政务中心、孙村、唐冶片区、大学科技园等重点片区开发建设。三是加快老城提升步伐。重点抓好大明湖、四大泉系、泉城广场周边、小清河综合治理等重大项目建设，实施城市快速公交一期工程，搞好城市近100条主次干道建设、改造。四是全面提升城市管理水平。继续深化城市管理体制改革，在落实市区权责、完善城管综合执法、提高应急服务能力上迈出更大步伐。五是深入开展城乡环境综合整治。巩固街巷道路、违规建设、交通秩序等整治成果，重点在城市村居、重点区域、村镇容貌等方面加大整治力度，实现城乡面貌较大改观。六是加强城市生态环境保护。全面落实生态城市建设规划，强化重点污染防治，加大垃圾和污水处理力度，显著改善大气环境和市内河道水体质量。七是全面提高城市运营水平。整合利用城市各类资源，提高土地集约利用水平，严格执行投融资各项制度，保障重点建设工程顺利实施。

　　(五)加快产业和基础设施延伸，促进区域互动协调发展。按照统筹规划、分类指导、突出特色、优势互补的思路，努力形成区域发展新格局。一是加快推动“四沿”开发。加强经十路两侧纵深开发，启动铁路沿线和小清河改造工程，加快实施鹊山龙湖一期工程。二是加快大交通体系建设。构筑以中心区为核心、辐射远郊、四通八达的大交通网络。东向贯通章丘大道与泉城路，西向加快济菏高速平阴段建设，南向拓宽改造103省道，完成104国道改造，畅通城市南出口，北向加快“五路过河”工程实施，完成省道248线改造。三是完善统筹协调机制。发挥财政资金杠杆作用，加大转移支付力度，统筹建设城乡基础设施和公共服务设施。以市场配置为基础，积极引导产业科学布局、合理流动。进一步完善激励导向政策，鼓励各发展主体发挥比较优势，加快发展。四是推进南部山区保护性开发。按照整合资源、优化布局、加强监管的原则，加快制订南部山区保护性发展总体规划和专项规划，兼顾生态保护和改善人民生活的需要，大力发展林果业，加快发展旅游业，搞好村庄整合试点。

　　(六)加强重点领域改革和对外开放，增强经济发展自身活力。坚持改革开放协调推进，不断提高市场化程度和对外开放水平。一是深化企业改革。按照“做大做强一批、放开搞活一批、退出市场一批”的思路，加快推进国有经济布局和结构战略性调整，积极发展混合所有制经济，基本完成市属国有(集体)企业改革改制任务。二是推进事业单位改革。制定出台事业单位改革相关配套政策，按照先易后难、稳步实施的原则，积极推进行政支持类、公益类、经营类事业单位改革改制。三是全方位推进对外开放。将引进外资与促进产业结构升级、提高技术水平和研发能力相结合，积极引导境外和市外资金投向高新技术产业、先进制造业、现代服务业、现代农业，参与国有资产重组。转变外贸增长方式。优化出口商品结构，加强对重点大户的协调服务，努力培植一批新的出口企业，扩大附加值高、科技含量高产品出口。推进多领域对外合作。继续实施“走出去”战略，鼓励有比较优势的企业境外投资，推动海外承包工程与劳务输出密切结合。认真落实推进出口各项政策，推进“大通关”工程，努力开辟新的国际航线。

　　(七)突出项目带动和消费拉动，着力培植新的经济增长点。优化投资结构，启动新兴消费热点，实现全社会固定资产投资1100亿元，力争投资率突破50%。一是强化大项目支撑。储备策划一批关联带动力强、支撑作用明显的重点项目，着重抓好新开工的千万元以上项目，力争一大批重点项目年内竣工并发挥效益。二是优化投资结构。把保持投资强度与优化投资结构统一起来，重点加大高新技术、先进制造业、新农村建设、基础设施、社会事业、生态环境等领域投入力度，坚决遏制低水平建设和耗能高、污染重、资源浪费项目。三是拓宽融资渠道。积极争取境外资金，大力吸引国内资金；积极争取政策资金和信贷资金，大力吸引社会资本和境外资本；招商引资额达到560亿元，占固定资产投资比重突破50%。四是突出消费拉动作用。努力增加城乡居民收入，提高中低收入阶层保障和收入水平，增强消费预期；积极开拓农村消费市场，引导新的消费热点和领域；加强市场监管，严厉打击制假售假和商业欺诈行为，努力改善消费环境。

　　(八)完善科教人才工作机制，提升城市自主创新能力。以建设创新型城市为目标，努力增强科技、教育、人才对经济社会发展的支撑作用。一是加快科技创新步伐。把握关系产业结构调整的重点领域和关键环节，加快培植一批具有自主知识产权与核心技术的企业技术中心，启动实施高新技术自主创新工程和节约型社会科技支撑体系工程，完善知识产权保护、风险投资、技术产权化三项制度，争取更多项目列入国家和省科技计划，在创新制度构建和促进产业发展上迈出新步伐。二是加快教育事业发展。强化政府义务教育责任，突出解决基础教育资源不均衡的问题，加强城乡统筹，推进教育公平。巩固完善“以县为主”农村义务教育管理体制，落实振兴农村教育行动计划，加大对“两免一补”投入。根据就业需求扩大职业学校招生规模，着力培养各类技能型适用人才。加快大学科技园和职业教育基地建设步伐，带动高等教育规模质量提升。三是加强人力资源建设。重点抓好专业人才、高技能人才和农村实用人才的培养。充分发挥留学人员创业园等载体作用，大力吸引留学人员和国内优秀人才来济创业。健全人才市场服务体系，完善人才资源市场配置和人才评价机制，提高人才资源对经济社会发展支撑力。优化人口结构，提高人口素质，积极推进人口资源向人力资源转变。

　　(九)更加关注和解决民生问题，扎实推进“和谐济南”建设。认真解决涉及群众切身利益的矛盾和问题，更加注重社会公平和公正，以文明城市创建活动为总抓手，加快推进“和谐济南”建设。一是大力促进就业再就业。认真落实各类扶持引导政策，重点解决好下岗失业人员再就业、农村劳动力转移和大学生就业工作，力争新增城镇就业13万人。二是加快完善社会保障体系。依法加强社会保险费征缴扩面和清欠工作，进一步扩大企业养老、失业、医疗、工伤、生育保险以及住房公积金覆盖面。完善社会福利和优抚安置政策，提高劳动保障执法维权能力，加快疾控中心建设和传染病医院迁建。三是切实关心困难群众生活。建立健全以城乡低保、农村五保等十项制度为主体的社会救助体系，切实关注妇女儿童、老年人、残疾人以及弱势群体，努力解决上学难、看病难和住房难等问题，加强食品药品安全整治工作。四是深入开展文明城市创建活动。坚持“为了人民创城、依靠人民创城”的方针，加强薄弱环节，推动文明创建活动不断深入。全面落实社会治安综合治理措施，努力维护社会稳定。高度重视信访工作，健全法律援助服务体系，引导群众以合法形式表达利益诉求。严格落实各级安全生产责任，坚决防止重特大安全事故发生。

　　(十)继续深化行政体制改革，加快政府职能转变步伐。加快政府职能转变，提高公共服务能力，努力建设人民满意政府。一是推进政府职能转变。加强经济调节，不断加大规划引导和政策扶持力度；强化市场监管，切实维护公平、公开和开放有序的市场经济秩序；规范社会管理，逐步健全政府行政调控与社会自治互动的管理网络；完善公共服务，着力提供经济性、社会性、制度性三大公共产品。二是改革行政管理体制。优化政府组织结构，减少行政职能交叉，理顺职责分工，推行电子政务，提高行政效率，降低行政成本。深化行政审批制度改革，简化和规范行政许可程序。继续推进政府资产管理制度改革，稳妥推进事业单位用人、分配、社会保障等综合配套改革。三是创新行政运行机制。完善公众参与、专家论证和政府决策相结合的科学决策机制，建立权责明晰的行政执法责任制和行政执法协调机制，推行科学的绩效评价机制和过错责任追究机制，完善政务公开制度，提高政府行为的透明度。四是加强廉政建设。抓好政风建设，坚决纠正损害群众利益的不正之风；强化对权力运行监督制约，促进领导干部廉洁从政；坚持从严治政，严肃查处违反政纪的案件，推进从源头上预防腐败。以《公务员法》实施为契机，以提高执政意识和行政能力为重点，努力提升公务员队伍素质。始终坚持“情为民所系、权为民所用、利为民所谋”，牢固树立科学发展观和正确政绩观，大兴求真务实之风，坚决反对形式主义，脚踏实地为群众办实事、做好事、解难题，全力维护群众利益。

　　各位代表，未来五年是我市全面推进科学发展、加快构建和谐社会的关键时期。我们要倍加珍惜来之不易的大好形势，更加紧密地团结在以胡锦涛同志为总书记的党中央周围，在省委、省政府和市委的坚强领导下，扎实工作，开拓进取，把握新机遇，全力开创各项工作新局面，为加快省会改革开放和现代化建设而努力奋斗！
